# Innovation in neurosurgery: less than IDEAL? A systematic review

**DOI:** 10.1007/s00701-017-3280-3

**Published:** 2017-08-06

**Authors:** I. S. Muskens, S. J. H. Diederen, J. T. Senders, A. H. Zamanipoor Najafabadi, W. R. van Furth, A. M. May, T. R. Smith, A. L. Bredenoord, M. L. D. Broekman

**Affiliations:** 1 Department of Neurosurgery, University Medical Center Utrecht, HP G03.124, PO Box 85500, 3508 GA Utrecht, The Netherlands; 2 Cushing Neurosurgery Outcomes Center (CNOC), Department of Neurosurgery, Brigham and Women’s Hospital, Harvard Medical School, Boston, MA USA; 3 Department of Neurosurgery, Leiden University Medical Center, Leiden, The Netherlands; 4 Department of Epidemiology, Julius Center for Health Sciences and Primary Care, University Medical Center Utrecht, Utrecht, The Netherlands; 5 Department of Medical Humanities, Julius Center for Health Sciences and Primary Care, University Medical Center Utrecht, Utrecht, The Netherlands

**Keywords:** IDEAL framework, Innovation, Neurosurgery, Meningioma, Ethics, Intracranial aneurysm, WEB device

## Abstract

**Background:**

Surgical innovation is different from the introduction of novel pharmaceuticals. To help address this, in 2009 the IDEAL Collaboration (Idea, Development, Exploration, Assessment, Long-term follow-up) introduced the five-stage framework for surgical innovation. To evaluate the framework feasibility for novel neurosurgical procedure introduction, two innovative surgical procedures were examined: the endoscopic endonasal approach for skull base meningiomas (EEMS) and the WovenEndobridge (WEB device) for endovascular treatment of intracranial aneurysms.

**Methods:**

The published literature on EEMS and WEB devices was systematically reviewed. Identified studies were classified according to the IDEAL framework stage. Next, studies were evaluated for possible categorization according to the IDEAL framework.

**Results:**

Five hundred seventy-six papers describing EEMS were identified of which 26 papers were included. No prospective studies were identified, and no studies reported on ethical approval or patient informed consent for the innovative procedure. Therefore, no clinical studies could be categorized according to the IDEAL Framework. For WEB devices, 6229 articles were screened of which 21 were included. In contrast to EEMS, two studies were categorized as 2a and two as 2b.

**Conclusion:**

The results of this systematic review demonstrate that both EEMS and WEB devices were not introduced according to the (later developed in the case of EEMS) IDEAL framework. Elements of the framework such as informed consent, ethical approval, and rigorous outcomes reporting are important and could serve to improve the quality of neurosurgical research. Alternative study designs and the use of big data could be useful modifications of the IDEAL framework for innovation in neurosurgery.

**Electronic supplementary material:**

The online version of this article (doi:10.1007/s00701-017-3280-3) contains supplementary material, which is available to authorized users.

## Introduction

Today, it is unusual to perform neurosurgical procedures in most countries without access to an operative microscope, state-of-the-art neuro-navigational systems, or even hemostatic agents such as a bipolar electrocautery devices. In fact, technological innovation has been the hallmark of neurosurgery, and the vast majority of procedures that are currently considered routine would not be possible at all without innovation. However, not all innovation is an improvement over the technology it seeks to supplant. Evidence of patient outcome superiority is often lacking or non-existent in the real-time of innovation. In neurosurgical disease, low incidence and high burden may further hinder systematic evaluation of any new technique. Regardless of these difficulties, it is vital that new technologies and procedures undergo a strategic and ethical clinical introduction [9].

As surgical innovation does not typically follow the same introductory path as novel pharmaceuticals, the IDEAL Collaboration, formed by surgeons and methodologists, introduced the IDEAL (Idea, Development, Exploration, Assessment, Long-term follow-up) framework in 2009 and have published several updates since [16, 23, 47, 48, 65]. The goal of the collaboration is to improve surgical research, especially research surrounding innovation, and to overcome obstacles and methodological problems inherent to surgery [29, 47].

The IDEAL framework describes five stages through which interventional therapeutic innovations typically pass, together with the characteristics and study design of each stage (Table 1, adapted from McCulloch et al.) [16, 23, 47, 48, 65]. Any study involving non-human pre-clinical assessment of a novel technique, including simulator or animal studies, is regarded as stage 0. Stage one describes a proof-of-concept study in the first human patient. Stage 2a consists of a prospective study in up to 30 patients conducted by surgeons responsible for the earlier stage(s). Involving surgeons with no prior experience in a larger prospective study usually takes place in stage 2b to assess the learning curve and further develop the procedure. In stage 3, the procedure should be stable and is investigated in a randomized controlled trial (RCT) that compares outcomes of the innovative procedure with the gold standard. Assessment of rare and long-term outcomes takes place in stage 4 (Table 1) [29, 47].

**Table 1:** Overview of the IDEAL framework, adapted from McColloch [47]

Purpose	1: Idea	2a: Development	2b: Exploration	3: Assessment	4: Long-term study
	Proof of concept	Development	Learning	Assessment	Surveillance
Number and types of patients	Single digit, highly selected	Few; selected	Many; may expand to mixed; broadening indication	Many; expanded indications	All eligible
Number and types of surgeons	Very few; innovators	Few; innovators and some early adaptors	Many; innovators, early adaptors, early majority	Many; early majority	All eligible
Output	Description	Description	Measurement; comparison	Comparison; Complete information for non RCT participants	Description; audit; regional variation; quality assurance; risk adjustment
Intervention	Evolving: procedure inception	Evolving; procedure development	Evolving; procedure refinement; community learning	Stable	Stable
Method	Structured case reports	Prospective development studies	Research database; explanatory or feasibility RCT	RCT with or without additions/modifications; alternative designs	Registry; routine database; rare case reports
Outcomes	Proof of concept; technical achievement; Disasters; dramatic successes	Mainly safety; technical and procedural success	Safety; clinical outcomes; short-term outcomes; patient centered outcomes feasibility outcomes	Clinical outcomes; middle-term and long-term outcomes; patient-centered outcomes; cost-effectiveness	Rare events; long-term outcomes; quality assurance
Ethical approval	Sometimes	Yes	Yes	Yes	No

To assess whether the IDEAL framework has been used two different neurosurgical procedures were evaluated: an endoscopic endonasal approach for skull base meningiomas (EEMS) and the use of the Woven Endobridge (WEB device, ©Sequent Medical) for endovascular treatment of intracranial aneurysms. Traditionally, skull base meningiomas are resected using an open transcranial microscopic approach [36]. However, recently, EEMS has been introduced and has gained some traction in neurosurgical literature [36]. The WEB device is a new option for intracranial aneurysm treatment, consisting of an unfoldable, detachable metallic mesh that is placed into the aneurysm neck leading to flow disruption [35]. The WEB device was especially developed for bifurcation and wide neck aneurysms as an alternative to traditional clipping or coiling [35]. Since the two innovations, one a device and the other a procedure, are used in different fields of neurosurgery and were recently introduced, we chose these two as examples for neurosurgical innovations in general.

In this review, published literature on these two procedures was evaluated to assess whether they were introduced according to the stages of the IDEAL framework.

## Methods

### Search strategy and paper selection

**Fig. 1:**
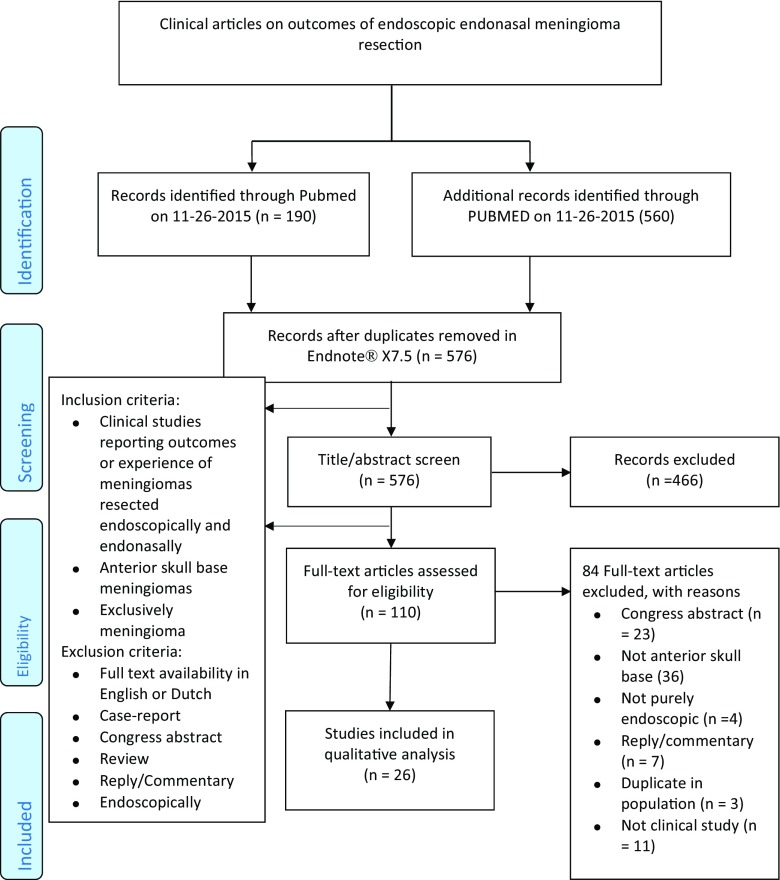

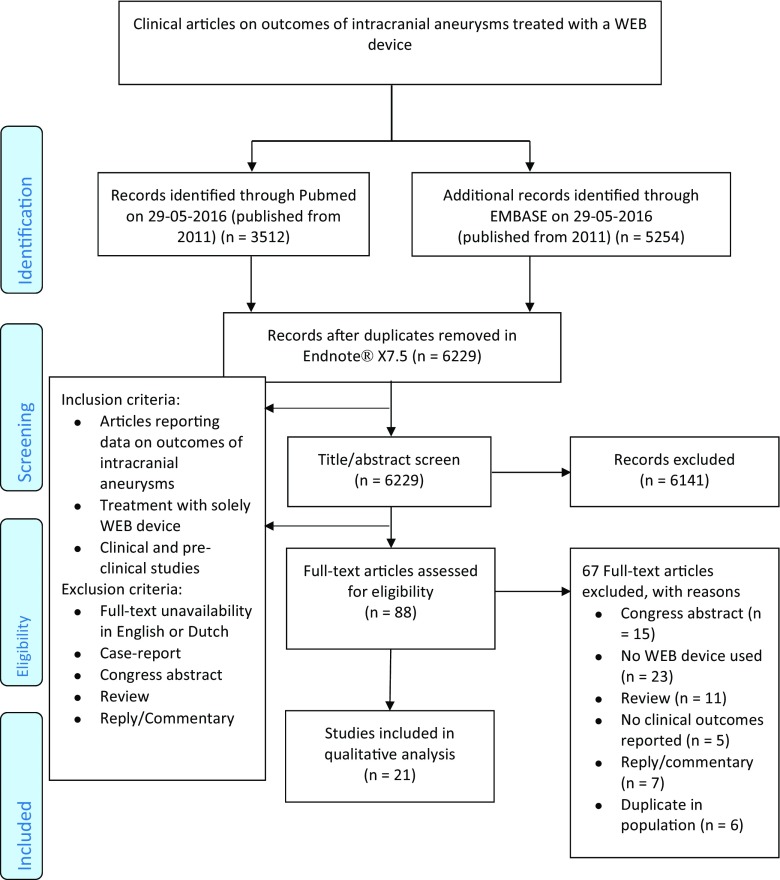
**a** Flowchart of the study selection process of included articles on endoscopic endonasal meningioma resection. **b** Flowchart of the study selection process of included articles on WEB devices

This systematic review was conducted in accordance with the Preferred Reporting Items for Systematic reviews and Meta-Analyses (PRISMA) statement [50]. The literature search for EEMS was conducted in PubMed and Embase up to 26 November 2015, using the following keywords: endoscopy, neurosurgery, endo- and transnasal and meningioma. The search strategy is provided in Supplemental Digital Content Table 1a. This search strategy resulted in 576 unique papers. In addition, bibliographies of the included papers were screened for relevant papers. For WEB devices, a search was conducted in the same search engines on 29 May 2016 using the keywords: WEB device, endovascular treatment, and intracranial aneurysm as depicted in Supplemental Digital Content Table 1b. This resulted in 6229 articles. These papers were supplemented by hand searching of the bibliographies of the papers retrieved by the electronic search. This review was restricted to published data. Only papers written in English, Dutch, French, or German were considered for this review. The search was not limited by date of publication. Titles and abstracts of retrieved citations were screened by two authors, and potentially suitable studies for EEMS were read in full by IM and SD and for WEB devices by IM and JS. We included papers that solely focused on EEMS as depicted in Fig. 1a [50]. For WEB devices we included papers reporting outcomes of treated aneurysms as described in Fig. 1b [50]. Disagreements were solved by reviewer consensus.

### Study assessment

Relevant studies were reviewed in full text to determine whether they could be classified according to an IDEAL stage by two authors (IM and SD for EEMS and IM and JS for WEB devices) [47]. The following criteria were used to classify studies according to the five stages. Pre-clinical studies were classified as stage 0, and proof of principal in one patient was regarded as stage 1 if informed consent had been obtained [47]. Studies were classified as stage 2 if ethical approval for a prospective study and informed consent for an innovative procedure from included patients had been obtained. Studies with up to 20 patients were classified as stage 2a and those with more than 20 patients as stage 2b. Studies that compared EEMS or WEB devices with the current gold standard in a prospective fashion were regarded as stage 3. As an RCT may not have been feasible for ethical or pragmatic reasons, we also evaluated studies with different designs [16, 23]. Long-term follow-up studies were categorized as stage 4. In addition to study design, ethical approval and informed consent, all studies were evaluated for reporting surgical or radiological outcomes for EEMS and WEB devices studies, respectively. Disagreements were solved by consensus discussion.

## Results

For EEMS, 576 abstracts and titles were screened, 110 were examined full text, and 26 papers were included (Fig. 1a) [1, 8, 12, 13, 17, 19–21, 24–26, 30, 32, 34, 37, 38, 52–55, 62, 68, 70–73]. Two cadaveric studies were categorized as stage 0 [12, 30]. No studies were categorized as stage 2, as none of the included studies reported outcomes of a prospective study with adequate informed consent [1, 8, 13, 17, 19–21, 24–26, 32, 34, 37, 38, 52–55, 62, 68, 70–73]. Even though four studies compared EEMS with an open transcranial approach, they did not do this in a prospective fashion, and no RCTs could be identified [8, 19, 20, 52]. Furthermore, there were no studies that examined long-term outcomes, and therefore no studies were categorized as stage 4 (Table 2). All other studies could not be categorized into an IDEAL stage.

**Table 2:** IDEAL Framework recommendations and endoscopic endonasal meningioma surgery

Author (year of publication)	Participants (N=)	Ethical approval for prospective study	Informed consent for innovative procedure	Described surgical outcome	Randomized controlled trial	Awarded IDEAL stage
Cavallo et al. (2005) [12]	0	NA	NA	NA	NA	0
Jacquesson et al. (2015) [30]	0	NA	NA	NA	NA	0
Alexander et al. (2010) [1]	1	N	N	Y	N	None
Bowers et al. (2011) [8]	27	N	N	Y	N	None
Chowdhury et al. (2012) [13]	6	N	N	Y	N	None
Cook et al. (2004) [17]	3	N	N	Y	N	None
De Almeida et al. (2015) [19]	20	N	N	Y	N	None
De Divitiis et al. (2008) [20]	51	N	N	Y	N	None
De Divitiis et al. (2008) [21]	11	N	N	Y	N	None
Fernadez-Miranda et al. (2012) [24]	1	N	N	Y	N	None
Gadgil et al. (2013) [25]	5	N	N	Y	N	None
Gardner et al. (2008) [26]	35	N	N	Y	N	None
Julian et al. (2014) [32]	1	N	N	Y	N	None
Khan et al. (2014) [34]	46	N	N	Y	N	None
Koutourousiou (2014) [37]	75	N	N	Y	N	None
Koutourousiou (2014) [38]	50	N	N	Y	N	None
Mortazavi et al. (2015) [52]	27	N	N	Y	N	None
Ogawa et al. (2012) [53]	19	N	N	Y	N	None
Ottenhausen et al. (2014) [54]	20	N	N	Y	N	None
Padhye et al. (2012) [55]	15	N	N	Y	N	None
Prevedello et al. (2007) [62]	1	N	N	Y	N	None
Van Gompel et al. (2011) [68]	13	N	N	Y	N	None
Wang et al. (2009) [71]	7	N	N	Y	N	None
Wang et al. (2010) [70]	12	N	N	Y	N	None
Wang et al. (2015) [72]	1	N	N	Y	N	None
Webb-Myers et al. (2008) [73]	1	N	N	Y	N	None

*The Y (Yes)* means the study meets the IDEAL framework recommendations. *The N (No)* means the study did not meet the IDEAL framework recommendations. *NA* Not applicable

For WEB devices, 6229 abstracts and titles were screened, 88 articles were examined full text, and 21 papers were included (Fig. 1b) [2, 5, 10, 11, 14, 15, 22, 27, 33, 35, 41–43, 45, 56–60, 64, 69]. Preclinical studies using rabbit models were classified as stage 0 [22, 64]. One study that acquired informed consent for treatment of two patients was categorized as stage 1, but did not describe the clinical problem that needed a solution [35]. Two studies with ethical approval for a prospective study and informed consent of included patients were categorized as stage 2a [2, 44]. The studies with larger populations that reported the outcomes of the WEBCAST trial and the French observatory trial were categorized as stage 2b [57, 60]. All other studies could not be categorized into an IDEAL stage, and no studies were categorized as stage 3 or 4 as no comparison was made with other treatment modalities and no long-term outcomes were evaluated (Table 3).

**Table 3:** IDEAL framework recommendations and the WEB device

Author (year of publication)	Participants (N=)	Ethical approval for prospective study	Informed consent for innovative procedure	Described radiological outcome	Randomized controlled trial	Awarded IDEAL stage
Ding et al. (2011) [22]	24 (rabbits)	NA	NA	NA	N	0
Rouchaud et al. (2016) [64]	80 (rabbits)	NA	NA	NA	N	0
Ambrosi et al. (2015) [2]	10	Y	Y	Y	N	2a
Behme et al. (2015) [5]	52	N	N	Y	N	None
Caroff et al. (2014) [10]	6	N	N	Y	N	None
Caroff et al. (2015) [11]	98	N	N	Y	N	None
Clajus et al. (2016) [14]	108	N	Y^a^	Y	N	None
Colla et al. (2013) [15]	4	N	N	Y	N	None
Gherasim et al. (2015) [27]	10	Y	N	Y	N	None
Kabbasch et al. (2016) [33]	43	N	N	Y	N	None
Klisch et al. (2011) [35]	2	N	Y	Y	N	1
Lawson et al. (2016) [41]	23	N	N	Y	N	None
Lescher et al. (2016) [42]	22	N	N	Y	N	None
Liebig et al. (2015) [43]	47	N	N	Y	N	None
Lubicz et al. (2013) [44]	19	Y	Y	Y	N	2a
Papagiannaki et al. (2014) [56]	83	Y	N	Y	N	None
Pierot et al. (2013) [58]	33	N	N	Y	N	None
Pierot et al. (2015) [59]	45	N	N	Y	N	None
Pierot et al. (2016) [57]	51	Y	Y	Y	N	2b
Pierot et al. (2016) [60]	62	Y	Y	Y	N	2b
Van Rooij et al. (2016) [69]	32	N	N	Y	N	None

*The Y (Yes)* symbol means the study met the IDEAL framework recommendations. *The N (No)* symbol means the study did not meet the IDEAL framework recommendations

^a^Informed consent was only obtained in cognitively intact patients

## Discussion

The results of this systematic review demonstrate that both the endoscopic endonasal transsphenoidal approach for resection of skull base meningiomas and WEB devices were not introduced according to the IDEAL Framework. Not only could not all IDEAL framework stages be identified, some of the early pre-clinical studies (stage 0) were performed long after the description of the first-in-man studies (for EEMS) or after publication of prospective studies (WEB devices) [12, 22, 30, 64]. Perhaps unsurprisingly, only five clinical studies could be categorized into an IDEAL stage. WEB device studies followed the IDEAL Framework more closely than EEMS, but only up to stage 2b [2, 35, 45, 57, 60]. In addition, only six WEB device studies acquired ethical approval for a prospective study in line with the IDEAL framework [2, 27, 45, 56, 60, 61]. No study reported patient selection for EEMS compared to five WEB device studies [27, 41, 45, 57, 60]. Furthermore, no studies were categorized as stage 3 as no clinical study (of either procedure) was a prospective comparison with the gold standard or was an RCT.

We believe that this is not unique to these two procedures specifically or to neurosurgery in general. For instance, a study investigating literature on laparoscopic colonic polyp resection found that its introduction into widespread use also did not follow the stages and recommendations of the IDEAL framework [18].

The introduction of novel neurosurgical techniques that result in a paradigm change, i.e., the first endovascular treatment of aneurysms, could be introduced according to some predefined framework such as IDEAL. However, in reality, novel surgical techniques are often the result of small stepwise changes to existing approaches (e.g., EEMS and the transcranial approach to pituitary adenomas). This makes it challenging to introduce innovations as EEMS according to all requirements of the IDEAL framework. Adherence to the IDEAL framework might not only be challenging because of small stepwise changes of existing approaches but also because of a lack of a universally accepted definition of neurosurgical innovation in general.

A major change in endonasal surgery was the introduction of the endoscope, in particular for pituitary adenomas [31]. With expansion of endoscopic technique and experience, a wider spectrum of tumors became resectable through the endonasal approach. However, in retrospect, one could argue that EEMS is indeed a valuable alternative to a classic craniotomy for specific indications.

The WEB device is also example of expanding endovascular experience, and because of new endovascular devices a wider array of pathologies is treatable. Compared to EEMS, WEB devices were studied in a prospective fashion with patient informed consent [2, 45, 57, 60]. However, the WEB device is already used clinically despite lack of comparison with other treatment options (a stage 3 study) [11, 14, 69]. The important question is whether this new technique could have been rigorously compared to established techniques prior to wide-spread adoption.

Overall, this review suggests that neurosurgical innovation (at least for the two procedures evaluated here) has not historically followed the IDEAL framework. On the one hand, this could simply be caused by a lack of awareness of the framework. On the other hand, a different distinct possibility for this could be related to feasibility. The IDEAL collaboration recognizes that, to improve the quantity and quality of surgical research, these proposals/recommendations would have to be practical and adapted to the process of innovation [47]. Indeed, the IDEAL Collaboration supports several recommendations for specific (alternative) study designs and reporting standards at different stages of the framework [16, 23, 47]. These alternatives could contribute to the quantity and quality of neurosurgical research.

At the innovation stage (stage 1), the recommendations include online registries for first-in-man innovations. No reports on the entry of a study in a registry were found in our review. Often in neurosurgery innovations take place in an acute setting, and only in retrospect is there clarity with regards to the innovation itself. However, it is possible that future innovations could be entered in a registry, especially in the case of new devices like the WEB device. Registries could help reduce positive reporting bias inherent to new innovations. Reports of both successes and failures of new technology are useful for ethical innovation [51].

At the second development stage recommendations include: prospective development studies, protocol and study registries for prospective development studies in surgery and development of agreed reporting standards and definitions for key outcomes [29, 47]. These recommendations were not met for the introduction of EEMS and by only four studies for WEB devices [2, 45, 57, 60].

Again, not all of these recommendations may be possible in neurosurgery. However, protocol and prospective study registries are feasible in the neurosurgical field and could help ensure that clinical results of all patients are transparent and methodologically sound. Furthermore, novel techniques could be reported using professionally accepted reporting guidelines for prospective (and if inapplicable, retrospective) studies that favor clear interpretation of the study design and study results. Also, open comparison of individual studies and applicability of the reported outcomes would be useful. Key patient-centered outcomes for various pathologies result in research with comparable and clinically meaningful results.

All studies described the surgical outcomes, and this is outstanding. One next step could be to unify informed consent and outcomes reporting, which should include both positive and negative findings, for emerging innovative procedures. Furthermore, one could argue this process should be done in a more uniform manner across the neurosurgical field. One method might be the use of centralized regulation as seen with medical device approval by the Food and Drug Administration (FDA) [39, 40]. Alternatively, institutions or neurosurgical societies could create guidelines for reporting of trial registration, prospective design and patient registries, effectively following the IDEAL framework to a certain extent [48]. Nevertheless, informed consent and ethical approval for a prospective study are, we believe, something that should always be feasible when evaluating a new neurosurgical procedure.

No prospective randomized studies or RCTs, the ‘default option’ at the third or exploration stage of the IDEAL framework, were identified [47]. This may be one area of the IDEAL framework that is not completely feasible in all types of neurosurgical innovation. As discussed, innovation occurs by incremental but gradual changes over a prolonged period of time, and an RCT may not be the preferred study design for numerous reasons: (1) It is ethically challenging and practically impossible to compare EEMS to an open approach as the endonasal approach is not applicable to all patients; (2) the number of patients with skull base meningiomas is relatively small, which makes it difficult to recruit enough patients for proper statistical analyses; (3) the difference in outcomes between an open and endonasal approach might be small and therefore difficult to prove, especially with point 2 in mind; (4) there could be a lack of clinical equipoise; (5) surgeons might not be willing to participate because of personal treatment preference or experience [46]; (6) surgeons have different skill levels; (7) the location, extent and size of meningiomas vary, complicating inter-patient comparability and randomization, again complicated by point 3; (8) concomitant factors can change during the trial, e.g., innovation in anesthesiology and perioperative care [6, 49]; (9) improvement of endoscopic endonasal meningioma surgery is a constantly evolving process with differences in every center, which contributes to the often reported difficulty in standardization for innovative surgical procedures, it is inefficient to conduct an RCT for every incremental technological advance, and the incidence of these lesions is quite low [6]. For these reasons, a “classical” RCT in low-volume, highly complex cases as with skull base meningioma resections or similar procedures might not be feasible. However, the IDEAL collaboration endorses various alternatives to this trial design at the third stage. These include case-matching studies and controlled interrupted-time series designs, but also modified RCTs with Baysean modifications to recruitment, randomization or analysis [47]. These study designs might be useful in neurosurgical innovation. Especially the introduction of prospective research databases and collaborative studies, endorsed by the IDEAL collaboration, seem valuable for low-volume, highly complex surgeries such as skull base meningioma resections. Also, the recommended additions to the RCTs that include learning curve evaluation, quality control and compliance measures could be feasible and helpful for innovations as EEMS.

Even though an RCT for WEB devices could be challenging, especially because of the above-mentioned reasons 3–9, an RCT is possible and could have been conducted prior to wide-spread European adoption [35]. However, in the absence of a traditional RCT, a Baysian RCT or registry could have also been helpful to establish its efficacy and safety. In fact, application of all stages of the IDEAL framework in a more strategic fashion could be possible in technological innovations like the WEB device. To date, the WEB device appears to be efficacious and safe, but a more rigorous and transparent process for the introduction of this type of technology could potentially help prevent deleterious outcomes, as seen with the Poly Implant Prothèse (PIP) breast implants and metal-on-metal hip prostheses [4, 28, 67]. Currently, proof of safety and efficacy is required by the FDA for class III devices (the most invasive devices), but this is not standardized [39, 40]. Therefore, a change in regulation that results in a closer adherence to the IDEAL framework could lead to a more uniform implementation [48].

At the fourth or long-term study stage, the emphasis is on rare and long-term outcomes. We did not identify any (stage 4) studies reporting long-term outcomes of EEMS or WEB devices. We believe that in addition to a closer adherence to the ‘IDEA’ part of the IDEAL framework, attention to the long-term outcomes of innovations such as EEMS or WEB devices would greatly benefit innovation in neurosurgery. Registries are an appropriate study design for this purpose, although representativeness of the data is a potential limitation. Efforts made to ensure that data entry is complete help strengthen the representativeness of the registry [47]. Reporting fatigue can compromise comprehensive data collection; therefore, the development of concentrated, outcome relevant registries are optimal. Also, the use of registries with patient informed consent for surveillance of specific established techniques in neurosurgery is desirable, especially for use of new materials like the WEB device.

In general, innovation in low-volume, highly complex (neuro)surgical cases might benefit from alternatives to traditional RCTs. For example, in a “cohort multiple RCT,” some, but not all, patients are randomly assigned to a specific treatment and are followed up regularly over time, blending a RCT with an observational study with some of their respective benefits [7, 63, 66]. A potential stage 3 study on a low-volume, highly complex surgical innovation could include the following: (1) patient informed consent; (2) ethical approval; (3) strict definition (and registration) of indications for treatment; (4) prospective observational design; (5) registration in a trial registry; (6) random allocation of a standard treatment group or the well-defined innovative procedure; (7) regular follow-up on relevant outcomes for patients; (8) reporting of all outcomes; (9) collaboration of multiple centers.

This, however, does not address the issue of which innovative procedures merit such a study. “Big data” could fill the gap with regard to identification of trial-worthy innovations. The use of the electronic medical record, the digitization of patient outcomes and the computational capacity now available to the typical researcher have opened the door to detailed and comprehensive analysis of pre-trial data. Indeed, these types of large data sets could become a new level of evidence in and of itself if an RCT is not feasible [3].

## Conclusion

The introduction of EEMS and WEB devices did not follow the stages as described by the IDEAL framework. The introduction of WEB devices followed the IDEAL Framework more closely, but only up to stage 2b. We believe this is not unique to neurosurgery or to these techniques, and it simply may not be feasible to follow this framework in its current iteration for all types of innovation. Despite this, informed consent, ethical approval and rigorous outcomes reporting are important elements of the IDEAL framework, which could serve to improve the quality of both experimental and alternative neurosurgical study designs. Alternatives to traditional RCTs and the use of “big data” could be useful modifications of the IDEAL framework. We believe that neurosurgical innovation and research could be improved by following a framework such as (a modified version of) IDEAL. This would improve evidence-based practice and potentially patient outcomes. After all, methodologically sound prospective studies, which require informed consent, ethical approval and equipoise, are feasible in neurosurgery.

## Electronic supplementary material

ESM 1 (DOCX 64 kb)
