# Supplementary material for: Innovation in neurosurgery: less than IDEAL? A systematic review
Source: Acta Neurochir (Wien). 2017 Aug 6;159(10):1957–66. doi: 10.1007/s00701-017-3280-3 (PMC5590028; doi:10.1007/s00701-017-3280-3)
Supplement: Supplementary file 1 — (DOCX 64 kb) [file 701_2017_3280_MOESM1_ESM.docx]

| PubMed | ((endoscopic*[Title/Abstract]) OR endoscopy[MeSH Terms]) AND ((((neurosurg*[Title/Abstract]) OR surg*[Title/Abstract]) OR Neurosurgical Procedures[MeSH Terms]) OR neurosurgery[MeSH Terms]) AND (((endonasal*[Title/Abstract]) OR transnasal*[Title/Abstract]) OR transsphenoidal*[Title/Abstract]) AND ((meningiom*[Title/Abstract]) OR meningioma[MeSH Terms])) |
| --- | --- |
| Embase | ('endoscopy'/exp OR endoscop*:ab,ti) AND ('neurosurgery'/exp OR neurosurg*:ab,ti OR surg*:ab,ti) AND (endonasal*:ab,ti OR transnasal*:ab,ti OR transsphenoidal*:ab,ti) AND ('meningioma'/exp OR meningi*:ab,ti) |

Supplementary Table 1a: search strategy for endonasal meningioma resection

| PubMed | Search (((((WEB[Title/Abstract]) OR Woven Endobridge[Title/Abstract])) OR (((("Endovascular Procedures"[Majr:NoExp]) OR "Embolization, Therapeutic"[Majr:NoExp])) OR ((((endovascular[Title/Abstract]) OR intravascular[Title/Abstract])) AND ((((((((technique*[Title/Abstract]) OR procedur*[Title/Abstract]) OR treatment[Title/Abstract]) OR surgery[Title/Abstract]) OR therapy[Title/Abstract]) OR flow disrupt*[Title/Abstract]) OR Embolization[Title/Abstract])))))) AND ((((((aneurism*[Title/Abstract]) OR aneurysm*[Title/Abstract])) AND (((((cerebral[Title/Abstract]) OR ruptured[Title/Abstract]) OR unruptured[Title/Abstract]) OR brain[Title/Abstract]) OR intracranial[Title/Abstract]))) OR intracranial aneurysm[MeSH Terms]) Filters:Publication date from 2011/01/01 to 2017/01/01 |
| --- | --- |
| Embase | (('web':ab,ti OR 'woven endobridge':ab,ti) OR ((endovascular:ab,ti OR intravascular:ab,ti) AND (technique*:ab,ti OR procedur*:ab,ti OR treatment:ab,ti OR surgery:ab,ti OR therapy:ab,ti)) OR ('endovascular aneurysm repair'/exp OR 'neurovascular embolization device'/exp OR 'device embolization'/exp OR 'artificial embolism'/exp)) ->8 AND (((aneurism*:ab,ti OR aneurysm*:ab,ti) AND (cerebral:ab,ti OR ruptured:ab,ti OR unruptured:ab,ti OR brain:ab,ti OR intracranial:ab,ti)) OR 'brain artery aneurysm'/exp OR 'intracranial aneurysm'/exp) AND [embase]/lim AND [2011-2016]/py |

Supplementary Table 1b: search strategy for WEB devices
